# Evaluating the outcomes of pluripotent stem‐cell‐derived photoreceptor transplantation in retinal repair

**DOI:** 10.1111/febs.70127

**Published:** 2025-05-10

**Authors:** Darin Zerti, Birthe Dorgau, Evelyne Sernagor, Lyle Armstrong, Majlinda Lako, Gerrit Hilgen

**Affiliations:** ^1^ Department of Biotechnological and Applied Clinical Sciences University of L'Aquila Italy; ^2^ Biosciences Institute Newcastle University Newcastle upon Tyne UK; ^3^ Department of Applied Sciences Northumbria University Newcastle upon Tyne UK

**Keywords:** photoreceptor, pluripotent stem cell, retinal neurodegeneration, transplantation

## Abstract

In recent decades, numerous research groups have focused on restoring visual function through the transplantation of stem cells into animal models of retinal neurodegeneration. Significant advancements in surgical techniques, the maturation of donor cells, and the production of cell suspensions, along with ensuring proper synaptic connectivity with the host environment, are key considerations for the potential implementation of this strategy in clinical practice. In this review, we summarize the latest progress in the transplantation of stem cell‐derived photoreceptors, emphasizing the outcomes related to visual function observed in the used animal models. Additionally, we analyze the various methods of stem cell differentiation and the surgical techniques selected for transplanting these photoreceptor precursors. Finally, we report on functional assessments from recent studies to highlight the considerable potential of stem cell‐derived photoreceptor transplants as a therapeutic approach for retinal degenerative diseases.

## Graphical abstract

Transplantation of stem cell‐derived photoreceptors in retinal repair. We summarize methods for differentiating stem cells and describe surgical techniques for transplanting photoreceptor precursors to restore visual function (A). The effectiveness of these techniques is assessed with behavioral (B) and *in vitro* assays (C) using mouse models that mimic retinal degenerative diseases. The artwork was adapted from Servier Medical Art (https://smart.servier.com/), which is licensed under CC BY 4.0 (https://creativecommons.org/licenses/by/4.0/).

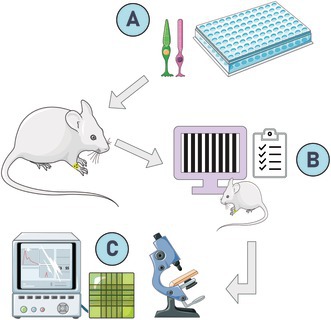

**Abbreviations**
AMD: age‐related macular degeneration
ChR: Channelrhodopsin
FDA: Food and Drug Agency
GFP: green fluorescent protein
hESCs: human embryonic stem cell
iPSCs: induced pluripotent stem cell
IRDS: inherited retinal diseases
LCA: Leber Congenital Amaurosis
ONL: outer nuclear layer
PR: photoreceptor receptor
RO: retinal organoid
RP: retinitis pigmentosa
RPCs: retinal progenitor cells
RPE: retinal pigment epithelium
VEGF: vascular endothelial growth factor

## Introduction

Diseases affecting the retina, the light‐sensitive extension of the central nervous system lining the back of the eye, are the most frequent causes of blindness worldwide. Photoreceptor cells are specialized neurons vulnerable to many retinal degenerative diseases. Two of the most common ones are retinitis pigmentosa (RP) and age‐related macular degeneration (AMD). It is estimated that AMD will affect 288 million people by 2040 [1, 2, 3], while RP will affect about 2.5 million people worldwide [2, 4]. These numbers are projected to increase dramatically in the next decades, posing important social and economic concerns. RP consists of a group of inherited retinal diseases due to genetic defects that damage photoreceptor cells directly or cause dysfunction of retinal pigment epithelium (RPE) cells [5]. AMD is the leading cause of visual impairment and blindness in the aging population. It is caused by a combination of genetic, lifestyle, and environmental risk factors [6] and results from progressive deterioration of the photoreceptor cells in the macula [7, 8]. While AMD first affects cones, leading to loss of central vision, RP first affects rod photoreceptors in the periphery, causing night blindness and tunnel vision. However, later on, cones also degenerate, leading to partial or complete blindness. Therefore, vision is ultimately lost in these conditions through a final common pathway of photoreceptor loss [5].

No effective treatment is available to restore vision once retinal cells have been lost. Early therapies included repeated intravitreal injections of antibodies to block the vascular endothelial growth factor (VEGF) like Bevacizumab and Ranibizumab [9]. The intravitreal injection of anti‐VEGF drugs represents the gold standard therapy for patients affected by the wet (exudative or neovascular) form of AMD, which accounts for around 10% of all AMD cases [10]. This therapeutic approach has many limitations due to the repeated intravitreal injections [11], in addition to an incomplete response to the treatment by many patients and side effects [12]. Furthermore, current anti‐VEGF therapies, considered a preventive approach for the neovascularization, can improve vision for a limited time in just 25–50% of patients [11, 12, 13, 14]. Recently, the US Food and Drug Administration (FDA) approved the complement factor 3 and 5 inhibitors to treat the geographic atrophy that represents the final stage of AMD [15, 16]; these are, however, posed by the same issue of repeated injections into the eye. However, these approaches are not effective at the end stage of the disease where most of the photoreceptors are lost, and thus alternative approaches need to be considered to restore sight. One option is implantable retinal prosthetic devices that aid in transmitting the signal to the brain via the optic nerve. Several prosthetic devices have improved visual perception (e.g., Argus II, Boston retinal implant or Alpha IMS) [17]. In cases where the retina has degenerated and lost its light‐sensitive photoreceptor cells but still retains other retinal cells, optogenetics or chemical‐based photoswitches could indeed be valid therapeutic approaches. Optogenetics refers to light‐sensitive proteins that, upon light activation [18], induce biological processes such as ion channel opening or closure, ion pumping across the cell membrane, or trigger intracellular signaling cascades. The optogenetic agent Channelrhodopsin (ChR) efficiently depolarizes neurons by opening non‐specific cationic channels through direct blue light stimulation. Halorhodopsins are light‐gated inward chloride pumps that cause hyperpolarization (inhibition) of the cell when triggered by strong yellow light [19]. However, these optogenetic tools require strong light sources to achieve sufficient membrane potential changes that can be translated into neural responses. Moreover, different opsin types need to be genetically targeted to different cell types to emulate intrinsic excitatory/inhibitory neural networks pertaining to retinal function. Thus, there is an urgent need to develop different therapeutic approaches that are cost‐effective and easily applicable to many patients.

To this end, several treatment approaches have been introduced to reverse, stop, or mitigate the course of retinal degeneration and to preserve the residual retinal cells. Unfortunately, in most cases, the death of photoreceptors remains unavoidable. Once photoreceptor cells have been lost, the only retina‐based strategies to restore vision are to replace dead cells or to support the surviving ones, with the final aim to restore light responses. The options for implementing these strategies include using neuroprotectant agents, such as natural compounds, and nano‐based drug delivery systems, including nano micelles, nanoparticles, or nanosuspensions [20]. The nanotechnologies aim to enhance the therapeutic efficiency, compliance, safety of eye therapeutic drugs, and elements administered via different routes. Some nanocarriers have recently received FDA approval and are currently available to treat eye diseases, including wet AMD, dry eye disease, keratitis, and uveitis [21]. Following the first FDA approval of ocular gene therapy in 2017 for Leber Congenital Amaurosis (LCA), an inherited retinal disease, there has been significant momentum in the field. This milestone has spurred numerous clinical trials exploring various techniques to treat a range of inherited retinal diseases (IRDs) [22].

In the field of regenerative medicine, numerous research groups are advancing stem cell‐based therapies aimed at treating retinal degenerative diseases. These therapies involve the transplantation of functional photoreceptor or precursor cells [23, 24, 25, 26, 27, 28]. The most significant advantage of photoreceptor transplantation is its potential to restore light sensitivity. This approach holds promise for patients with geographic atrophy due to AMD and those with other forms of photoreceptor‐related blindness, such as RP.

This review provides a comprehensive summary of recent advancements in the transplantation of stem cell‐derived photoreceptors, focusing on mouse models of retinal degeneration. We also highlight the functional outcomes of these transplants, focusing on their potential to restore vision.

## Stem‐cell‐based therapy

The main candidates for stem‐cell‐mediated retinal repair are retinal progenitor cells (RPCs), stem cell‐derived photoreceptors/photoreceptor precursors (PR/PRP), and RPE cells. Stem‐cell‐based therapy is considered an alternative approach to cure end‐stage retinal degeneration diseases, replace damaged retinal cells, or rescue and support the surviving retinal neurons. Many protocols have been established to produce PRs from stem cells over the years, mostly through the formation of retinal organoids (RO) [29, 30, 31]. With rapid advancements in the field, stem cell and RO technologies now provide a consistent, functional, and safe solution for patients requiring retinal transplants. These technologies also present a significant opportunity for therapeutic advancements in regenerative medicine.

One of the major challenges of stem‐cell replacement therapy is delivering the cells in a way that minimizes retinal impairment, while yielding successful engraftment and donor cell functionality. One surgical approach consists of the transplantation of cell suspension, which is achieved by injecting retinal cells in the subretinal space under microscope visual control [23, 24, 25, 27, 32, 33]. The main limitation of this approach is that the engrafted cells remain close to the injection site, in the subretinal space, and only limited numbers successfully integrate into the host retina, as reported by Zerti *et al*. [23, 24], Gasparini *et al*. [27] and Ribeiro *et al*. [25] and in some cases forming neuro‐rosettes, as firstly described by MacLaren *et al*. [34].

Photoceptor's precursors can be obtained from either human embryonic stem cells (hESCs) [23, 24] or induced pluripotent stem cells (iPSCs) [35] and fetal tissue [26, 36]. To increase the number of engrafted cells, enrichment of committed PR precursors PRPs—was introduced by MacLaren *et al*. [34], who transplanted cells with a green fluorescent protein (GFP)‐labeled rod precursors (Nrl‐gfp^+/+^ cells) in three mouse models characterized by slow and fast retinal degeneration. MacLaren *et al*. showed some incorporation of transplanted cells in the host Outer Nuclear Layer (ONL), a result attributed to the physical integration of donor photoreceptors. Additionally, the authors observed positive functional outcomes in rhodopsin‐null mice, as verified through either pupillometry testing or *ex vivo* extracellular field potential recordings from the ganglion cell layer. This was one of the earliest papers on photoreceptor transplantation. However, a few years later, researchers realized that many of the historical observations of donor tissue integration were actually due to material transfer between the transplanted cells and the host photoreceptors, rather than true integration [32, 37, 38, 39, 40, 41, 42].

Several research groups [23, 24, 25, 27, 43] replicated MacLaren's approach using a rodent model with partial or total retinal degeneration to transplant hESC and hiPSC‐derived PRPs, as first performed by Lamba *et al*. in 2008 [44, 45]. Donor cell engraftment was comparatively low in the first studies, but it improved over the years, as recently shown by Gasparini *et al*. [27]. As reported in Table 1, the authors demonstrated extensive incorporation of iPSC retinal organoid–derived human photoreceptors into the cone dysfunction mouse model Cpfl1, with about 20 000 cells engrafted into the host ONL [27]. In addition to incorporating into the host ONL over time, human cones also appeared to further mature *in vivo* until 26 weeks post‐transplantation, with numerous cones forming relatively well‐organized outer segments that were sometimes found to be joined to inner segments via a connecting cilium with the characteristic basal bodies at the ultrastructural level [25, 27]. Interestingly, these results were further corroborated by the presence of presynaptic terminal proteins, such as synaptophysin, Bassoon, and Ribeye, as also previously shown by Collin and Zerti [24]. Interestingly, Gasparini *et al*. [27] compared the transcriptional profiles of transplanted cells with those of age‐matched retinal organoids. Their results highlighted the crucial role of the host retina environment, showing that transplanted cells mature more extensively within the host retina compared to those maintained *in vitro*. In particular, the authors described an upregulation of genes related to pathways such as light perception and molecular and biological processes in the *in vivo*–matured cone samples compared to the *in vitro*, in addition to genes related to outer segment development [27].

**Table 1:** A list of relevant research articles based on similar preclinical animal studies utilizing stem cell therapy to restore vision. The papers were selected based on comparable methodologies, including precursor cell transplantation in mice followed by functional assays. A list of relevant research articles was published based on preclinical animal studies using stem‐cell‐based therapy. The papers were selected on the basis of the use of comparable methodologies, including precursor cell transplantation in mice followed by functional assays.

References	Injected cells	Animal model	Time after transplantation	Functional tests	Outcome
Pearson *et al*. (2012) [33]	200 000 mouse rod precursor cells	Gnat1^−/−^ mutant mice (6–8 weeks)	3–6 weeks	ERG, Water Maze, Optomotor response	Scotopic ERG response, improved light‐evoked behaviors
Santos‐Ferreira *et al*. (2016) [32]	200 000 ms cone‐like photoreceptors	Cpfl1 mutant mice (6–9 weeks old)	4 weeks	256 MEA	ON, OFF and ONOFF responsive RGCs
Zerti *et al*. (2021) [23]	150 000 human cone progenitor cells	C3H/HeNHsd‐Pde6brd1 mice (10–12 weeks)	3–4 weeks	4096 MEA, Light avoidance, Optomotor response	10 different responsive RGCs, improved light‐evoked behaviors
Ribeiro *et al*. (2021) [25]	500 000 human cone progenitor cells	rd1/Foxn1nu mice (11–14 weeks)	12–16 weeks	60 MEA, Light avoidance	10 different responsive RGCs, improved light‐evoked behaviors
Gasparini *et al*. (2022) [27]	150 000 human cone progenitor cells	Cpfl1 mutant mice (7–25‐week‐old)	10 and 26 weeks	256 MEA	ON, OFF and ONOFF responsive RGCs

Another photoreceptor manufacturing source is represented by retinal tissue sheets dissected from 3D‐retinas generated from human or mouse ESCs or iPSCs, in which a thin layer of cells is obtained and placed into the subretinal space. Several studies have demonstrated functional maturation after retinal sheets transplantation, with partial restoration of light responses in end‐stage retinal degeneration mouse models [46, 47, 48, 49], rats [28, 49, 50, 51] and in primates [49]. Despite the previously mentioned studies reporting visual improvement, these transplanted sheets appeared mostly disorganized, with many rosette formations due to the difficulty in maintaining good sheet orientation. Notably, the donor photoreceptors established synaptic connections to donor second‐order neurons rather than to host cells, although synaptic connectivity to host cells has been improved through the reduction of bipolar cells within the organoid transplant [52]. Hence, the efficacy of this approach is significantly reduced by the presence of donor interneurons in addition to glia, which reduce contact and connectivity between donor (mostly rod) photoreceptors and host bipolar cells [46, 48, 53].

Rodent models have historically been the most popular for retinal stem‐cell replacement studies due to their low cost, ease of genetic manipulation, and widespread availability. Among these models, the most widely used is the rd1 mouse model of RP (*C3H/HeNHsd*) homozygous for the retinal degeneration allele of the rod‐specific cGMP phosphodiesterase β6 subunit [23, 24, 25, 26, 32]. An interesting study by Barber *et al*. [54] compared the transplantation outcomes in six clinically relevant rodent models of retinal degeneration, including four models of RP (*Prph2* ^+/*Δ307*^, *Prph2* ^*rd2/rd2*^, *Rho* ^−/−^ and *PDE6β* ^*rd1/rd1*^), a model of Leber's congenital amaurosis (*Crb1* ^*rd8/rd8*^), and a model of stationary night blindness (*Gnat1* ^−/−^). Each model was characterized by varying rates of photoreceptor loss, ranging from a gradual decline (e.g., 10% loss over 12 months in the Gnat1^−/−^ model) to rapid degeneration (e.g., complete photoreceptor loss within 3 weeks in the PDE6βrd1 model). Notably, the *Crb1* ^*rd8/rd8*^ mice exhibited higher levels of engrafted cells, attributed to the complete disruption of the outer limiting membrane, which may facilitate, in some cases, the better integration and survival of transplanted photoreceptors. Importantly, at the time the study was published, the material transfer issue was unknown; hence, the conclusions presented by Barber *et al*. [54] should be considered carefully.

Another crucial factor in stem cell‐based retinal therapies is the cell dosage used in transplantation studies. Research by Zerti *et al*. [23] involved injecting 150 000 cone progenitor cells into the subretinal space. They observed a maximum of 1500 GFP^+^ cells after 3 weeks, indicating a relatively low survival or integration rate of the transplanted cells. Similarly, Gasparini *et al*. [27] injected 200 000 cells and found that approximately 10% of these cells were well incorporated into the host retina after 26 weeks. In a more recent study by Ho *et al*. [55], 175 000 cells per eye were injected into three different mouse models (C57BL/6J, Nrl^−/−^, and immune‐deficient NOD.Cg‐Prkdcscid Il2rgtm1Wjl/SzJ, also known as NOD‐SCID or NGS). The integration of human photoreceptors varied significantly across these models, with less than 5% integration in Nrl^−/−^ recipients and less than 1% in NSG recipients. This disparity in integration rates compared to the wild‐type C57BL/6J mice may be attributed to the breakdown of the outer limiting membrane in the mutant models, which can affect cell engraftment and survival. In contrast, promising outcomes were reported by Robin Ali's group [25]. They transplanted a higher number of sorted PRPs, specifically 500 000 cells. Four months post‐transplantation, the transplanted cell mass occupied approximately one‐third of the retina, demonstrating a higher level of integration and potential for restoring retinal function. These studies highlight the importance of optimizing cell dosage and understanding the interactions between transplanted cells and the host retinal environment to improve the effectiveness of stem cell‐based therapies for retinal degeneration.

In addition to rodent models, human PRPs have also been transplanted into non‐human primates, providing valuable insights into the potential and challenges of such therapies. Studies involving non‐human primates, such as those by Shirai *et al*. [56], Tu *et al*. [49], Chao *et al*. [57], and Lingam *et al*. [58], have demonstrated several important findings as described below.

Cell persistence, differentiation and anatomical challenges: Research by Lingam *et al*. [58] found that grafted human PRPs could persist and differentiate into cone photoreceptors, albeit at a low density. This indicates that the transplanted cells have the potential to integrate and develop into functional photoreceptors in a more complex retinal environment. Shirai *et al*. [56] and Chao *et al*. [57] observed anatomical disorganization within the retina following transplantation. These studies highlighted challenges related to the integration and organization of the transplanted cells, which can impact the overall efficacy of the therapy.

Overall, while transplantation of human PRPs in non‐human primates shows promise in terms of cell survival and differentiation, addressing issues related to anatomical integration and organization remains critical for advancing these therapies toward clinical application in humans.

Recent advancements in stem cell‐based therapies for retinal degeneration have also explored the use of canine models, which offer distinct advantages over rodent models. Dogs with naturally occurring mutations that mimic inherited retinal diseases provide a more relevant model due to the similarities in retinal anatomy and disease progression to humans [59]. Additionally, the larger size of canine eyes facilitates the optimization of surgical techniques and therapeutic approaches, making them more translatable to human patients. Ripolles‐Garcia *et al*. [60] transplanted hESC‐PRP into dogs with a form of rod‐cone degeneration caused by a nonsense mutation in the PDE6B gene (rcd1/PDE6B). This study was conducted at a late stage of retinal degeneration, approximately 29 weeks, when the retina had only two rows of surviving photoreceptors. The donor cell suspension was prepared from ROs isolated between days 104 and 151. The suspension was primarily composed of PRPs, which showed better engraftment in the degenerative canine retinas compared to wild‐type animals. This improved engraftment was attributed by the authors to the disruption of the outer limiting membrane associated with retinal degeneration compared to the wild‐type recipients, as previously reported by Stuck *et al*. [61]. The transplanted cells demonstrated the ability to develop into mature photoreceptors and establish synaptic structures. Notably, these cells were able to survive for up to 3–5 months post‐injection. The study assessed both conditions with and without systemic immunosuppression, providing insights into the role of immune response in cell survival and integration [60]. While the preclinical studies cited are indeed promising, several significant barriers and challenges must be addressed before stem cell replacement strategies can advance to human clinical trials. Key issues include:

Cell manufacturing

Scalability: Developing methods for large‐scale, reproducible production of high‐quality stem cell‐derived PRP is crucial [62]. This involves ensuring consistency in cell quality, functionality, and safety across different batches.

Standardization: Establishing standardized protocols for cell culture, differentiation, and processing is essential to meet regulatory requirements and ensure that the cells are suitable for clinical use.

GMP‐compliance: obtaining retinal tissue or RO to be used in therapeutic practice should be of top priority; hence, the entire process must pass Good Manufacturing Practice (GMP) and design guidelines [63, 64]. Allogeneic cells are used in the majority of the ongoing studies and clinical trials. As discussed by Cobb *et al*. [65] several aspects need to be achieved before bringing transplantation of PRPs to clinical trials. Among these, the possibility to use the bioreactors [66, 67] and automated cell culture systems [68] to create Xeno‐free conditions [69], reducing the time of hands‐on labor during RO production, thus reducing the cost of GMP certified staff, are paramount.

Donor cell dosage

Optimal Dosage: Determining the optimal number of donor cells required for effective therapeutic outcomes is challenging. Too few cells may not provide sufficient benefit, while too many could lead to complications or adverse effects.

Cell Survival and Integration: Ensuring that a sufficient proportion of transplanted cells survive, integrate, and function appropriately in the host retina is critical for successful outcomes.

Ideal host environment

Immunogenicity: Minimizing immune responses against the transplanted cells is essential for successful integration. This involves strategies such as:

Immunosuppression: Determining the need and extent of systemic or local immunosuppression to reduce the risk of rejection.

Immune‐privileged sites: Optimizing the transplantation environment to minimize immunogenic reactions, such as by targeting less immunogenic areas of the retina or using advanced techniques to make the host retina more compatible. Immunosuppression can be overcome by creating hPSC banks with known human leukocyte antigen (HLA) genotypes [70] as also discussed by Nair and Thomas [71], which are derived from a generic donor specifically selected to provide HLA matching to large portions of the overall population. Additionally, as recently reported by Lath *et al*. [72] significant research is ongoing with the aim to create a valid approach for improving immunological tolerance and decreasing immunogenicity, such as genetic engineering methods to change the cell surface antigens or use immunomodulatory substances [73].

Retinal Health: The state of the host retina at the time of transplantation can significantly impact the success of the procedure. Ensuring that the retinal environment supports cell integration and function is vital.

Long‐term safety and efficacy

Monitoring: Long‐term monitoring of transplanted cells and the host retina is necessary to assess the durability of the therapeutic effects and to detect any potential late‐onset adverse effects.

Functional Outcomes: demonstrating sustained improvements in vision and overall retinal function over time is critical for validating the efficacy of the therapy.

## Assessment of photoreceptor transplantation

The advancement of photoreceptor‐based cell replacement methods has yielded significant progress in restoring visual function. However, the successful integration of donor cells remains a critical challenge in transplantation. The loss of photoreceptors in the retina triggers a sequence of events, so‐called retinal remodeling, that permanently alters the structure of the neural retina. Retinal remodeling is progressive and occurs in all types of retinal degeneration [74, 75, 76, 77, 78]. The process of retinal remodeling is accompanied by glial scarring, which, together with the immune response triggered by xenotransplantation, becomes a significant obstacle to achieving successful engraftment [54, 79]. Researchers are actively exploring new approaches to overcome these obstacles and improve engraftment outcomes, using sensitive and comprehensive methods to validate successful engraftment into the retina. Structural assessment strategies such as immunohistochemistry and electron microscopy are often initially used to visualize the survival of the donor cells and newly formed synaptic connections. However, the gold standard for assessing successful functional repair of photoreceptor transplantation is to test with behavioral, reflex‐based, and electrophysiological approaches.

Behavioral tests are non‐invasive and allow for assessment at any time following the transplantation of stem‐cell‐derived photoreceptors. The most used test to assess if vision is successfully restored after transplantation of photoreceptors is the light‐avoidance test [23, 25, 80]. It is a quick and easy test. The experimental setup consists of a dark and bright chamber [81], allowing mice to move freely between them while the time in each chamber is monitored. Mice are nocturnal and usually avoid bright light. Blind mice would spend equal amounts of time in both chambers, while normal‐seeing mice would avoid the bright chamber as much as possible, except for occasional explorations. A variant of the light‐avoidance test, the Shuttle avoidance test [82], has also been used [48, 83]. Here, the test box is divided into two chambers by a dark wall with a small opening to enable the animal to move freely between them. A light signal is given as a warning for an incoming electric shock. To avoid the electric shock, the animal must move out of the chamber. The rd1 mouse model of RP was the backbone of all studies mentioned above. Rd1 mice show early onset and rapid retinal degeneration caused by mutations in *Pde6b*. The mice were aged 7–12 weeks, but the post‐transplantation time for assessment differed from 3 to 4 weeks [23, 80] over 2–3 months [48, 83] to 3–4 months [25], as reported in Table 1. Both tests can be affected by mice that are more active in terms of undirected exploration, anxiety, and locomotion in their behavior [84]. Nonetheless, all the above studies found that rd1 mice, after transplantation, stayed significantly longer than control animals in the dark chamber (avoiding light) or reacted to the light warning signal and left the chamber, respectively.

Reflex testing is also often used to assess visual abilities. The pupil light response (PLR) is driven by rods, cones, and intrinsically photosensitive retinal ganglion cells. This test is an automatic response that causes the pupil to contract in reaction to light, regulating the amount of light that enters the retina [85, 86]. Singh *et al*. [87] transplanted rod precursors into 8–12 weeks rd1 mice and assessed the visual function using Pupillometry [88] several weeks later. The study found functional improvement in the pupillary light response, not only proving the existence of light‐sensitive photoreceptors but also functional efferent connections to higher central targets. The optokinetic reflex or nystagmus (OKN) stabilizes an image on the retina during head movements [89]. The OKN is marked by slow and steady eye movements in line with the moving scenery. The optomotor reflex (OMR) refers to the movement of the head and body to this moving scenery. The animal is placed inside a drum with high‐contrast stripes and rotated to elicit and quantify the OKN and OMR. These days, a virtual cylinder is generated using computer monitors arranged in a square around the animal, presenting different spatial frequencies of sine or square waves with different contrasts at varying speeds [90]. Such a commercially available virtual optomotor system has been used to assess the successful reparation of visual function in transplanted mice. Both Zerti *et al*. [23] and Pearson *et al*. [33] used rod‐degenerated mouse models, rd1 and Gnat1^−/−^, respectively, and found that transplantation of cones improved head tracking (OMR) compared to control, untreated/sham‐treated animals. Typically, these experiments are assessed manually. The observer must decide whether the animal performed head tracking and in which direction, ideally without knowing the rotation and condition of the stimuli [90]. Such experiments are time‐intensive, depending on the subjective judgment of the observer and require experience. A faster and more objective method is to use automated tracking algorithms to quantify the OMR [91, 92] and OKN [93].

A more invasive method to functionally assess the success of photoreceptor transplantation is to use retinal explants for cellular neurophysiology. Conventional multielectrode array (MEA) devices [94], mERG [95], along with state‐of‐the‐art CMOS MEA array recording technologies [96, 97], have played a significant role in assessing functional retinal repair and successful engraftment of transplants in mice, thereby contributing to the success of photoreceptor‐based cell replacement methods. Following successful behavioral and reflex assessment, animals are selected for cellular neurophysiology studies. This review focuses explicitly on studies in rd1 mice, which examine successful photoreceptor transplantation using MEA technology, and compares the similarities of rescued light responses. Responses to light onset (ON) and offset (OFF), as well as to both (ON–OFF), are often named classical or conventional responses to light. The response length can be either transient or sustained. If a response does not follow the expected patterns, it is considered an unconventional response.

Zerti *et al*. [23] transplanted human embryonic stem‐cell‐derived cone photoreceptors into 10–12 weeks old C3H/HeNHsd‐Pde6brd1 mice. 3–4 weeks after transplantation, classical ON transient and sustained, OFF and ON–OFF light responses were recorded from retinal ganglion cells (RGCs) using a 4096‐channel high‐density CMOS MEA. Ribeiro *et al*. [25] transplanted hESC cones into 11–14 weeks rd1/Foxn1 and used a 120‐channel MEA to observe ON, OFF, and ON–OFF responses 3–4 months after transplantation. Both studies found identical recovered light response types in rd1 mice older than 12 weeks. Furthermore, both studies also discovered unconventional responses. Zerti *et al*. [23] described four different types; three showed prolonged OFF transient, sustained, and delayed responses but were suppressed by light (SBL) onset (SBL OFF transient, SBL OFF sustained, SBL OFF delayed), while the fourth had prolonged sustained ON responses but is suppressed by light offset/dark (SBD ON sustained). The study also reported a few cells that responded only during moving gratings but not to static flashes, suggesting some form of motion sensitivity could be potentially preserved. Ribero *et al*. [25] described several unconventional response patterns, including prolonged OFF responses, which are suppressed by light onset (OFF suppressed by light), ON suppressed by dark, and suppressed ON–OFF. Interestingly, the response patterns observed here are similar to those— SBL OFF transient, SBD ON sustained, and SBL OFF delayed— found in Zerti *et al*. [23]. Gasparini *et al*. [27] used a 256 MEA system and found conventional ON, OFF, and ON–OFF RGC responses. They were also able to map the receptive fields of RGCs restored by PR transplantation. Mandai *et al*. [48] and Matsuyama *et al*. [83] transplanted mouse iPSC retinal sheets into rd1/L7‐GFP mice at 7+ weeks old and tested responses to light using a 64‐channel MEA after 1.5–4 months. The majority of light responses were ON‐driven, but transient and sustained ON and ON–OFF responses were also observed. When the mGluR6 receptors (found in ON bipolar cells) were blocked, a different form of ON response was observed, called adapted ON, which included components of the OFF pathway. Similar crossover interactions were also seen in mGluR6 blocked ON–OFF responses, described as ON × OFF responses [83]. All these findings above underscore the need for further functional and structural investigation to better understand plasticity processes and *de novo* wiring in retinal pathologies.

Hyperactivity of RGCs in rd1 mice masks light responses in rd1 mice due to poor signal‐to‐noise ratio caused by rewiring of the neural retina [98]. The oscillatory activity can be suppressed by a gap junction blocker or potassium channel opener [99], which greatly helps to unmask potential light responses in transplanted rd1 retinas. However, gap junctions are crucial for retinal signal processing, including the transmission of rod‐mediated signals throughout the retina [100, 101], and blockade might affect certain pathways. Zerti *et al*. [23] demonstrated that low meclofenamic acid concentrations can be used in photoreceptor precursor transplanted rd1 mice to reduce oscillations and thus led to the discovery of a wider range of restored light responses, both conventional and unconventional.

## Conclusion and outlook

A combination of modern behavioral experiments, recent advancements in electrophysiology technology, and an understanding of retinal circuitry have helped uncover restored light responses from degenerated retinas. All the above studies have shown that transplanting photoreceptors into mice with retinal degeneration can partially restore their light responses. Though it is known that degenerated retinas can still exhibit residual light responses [78, 102, 103, 104] studies could show a correlation between the injected mass location and light‐responding RGCs. Notably, light‐driven RGCs typically represent less than 1% of the total active RGCs. In future studies, combining MEA recordings with *post hoc* immunofluorescence staining [105] would provide reliable correlation between engrafted cells and restored light responses. Another limiting factor for successful engraftment of donor cells is the immune system. Cloaking and scaffold strategies to foster engraftment are possible ways to improve the engraftment efficacy [106]. More sophisticated assessment methods, such as *in vivo* multiphoton imaging, allow access to cortical visual areas while mice freely behave in a visual test environment [107]. Such assessment methods allow long‐term behavior and visual functional performance testing without the need to sacrifice animals.

## Conflict of interest

The authors declare no conflict of interest.

## Author contributions

DZ, BD, ES, LA, ML, and GH drafted the manuscript and figures, edited, revised, and approved the final version of the manuscript.
